# The Complex World of Kynurenic Acid: Reflections on Biological Issues and Therapeutic Strategy

**DOI:** 10.3390/ijms25169040

**Published:** 2024-08-20

**Authors:** Trevor W. Stone, L. Gail Darlington, Abdulla A.-B. Badawy, Richard O. Williams

**Affiliations:** 1 The Kennedy Institute of Rheumatology, NDORMS, University of Oxford, Oxford OX3 7FY, UK; richard.williams@kennedy.ox.ac.uk; 2 Worthing Hospital, University Hospitals Sussex NHS Foundation Trust, Worthing BN11 2DH, UK; 3 Formerly School of Health Sciences, Cardiff Metropolitan University, Cardiff CF5 2YB, UK

**Keywords:** tryptophan, kynurenine, AHR, aryl hydrocarbon receptors, GPR35, hydroxy-carboxylic acid receptors, glutamate, NMDA, AMPA

## Abstract

It has been unequivocally established that kynurenic acid has a number of actions in a variety of cells and tissues, raising, in principle, the possibility of targeting its generation, metabolism or sites of action to manipulate those effects to a beneficial therapeutic end. However, many basic aspects of the biology of kynurenic acid remain unclear, potentially leading to some confusion and misinterpretations of data. They include questions of the source, generation, targets, enzyme expression, endogenous concentrations and sites of action. This essay is intended to raise and discuss many of these aspects as a source of reference for more balanced discussion. Those issues are followed by examples of situations in which modulating and correcting kynurenic acid production or activity could bring significant therapeutic benefit, including neurological and psychiatric conditions, inflammatory diseases and cell protection. More information is required to obtain a clear overall view of the pharmacological environment relevant to kynurenic acid, especially with respect to the active concentrations of kynurenine metabolites in vivo and changed levels in disease. The data and ideas presented here should permit a greater confidence in appreciating the sites of action and interaction of kynurenic acid under different local conditions and pathologies, enhancing our understanding of kynurenic acid itself and the many clinical conditions in which manipulating its pharmacology could be of clinical value.

## 1. Introduction

For more than 60 years after the discovery of the indolic amino acid tryptophan, one of its metabolites, kynurenic acid, was regarded as a biologically inactive by-product of tryptophan oxidation. This view persisted for several decades even though other metabolites were found, comprising the ‘kynurenine pathway’ from kynurenine to quinolinic acid and then nicotinamide (vitamin B3), producing the ubiquitous enzyme cofactor oxidised nicotinamide adenine dinucleotide (NAD+). The pathway was consequently considered to have evolved as a method for the de novo synthesis of NAD in a situation of dietary vitamin B deficiency. Without the kynurenine pathway, the basic biochemistry of life would be very different. 

It is now widely recognised that the kynurenine pathway (KP) plays fundamental roles in most tissues of the body and is altered in a variety of medical disorders. In most of these tissues and conditions, two aspects of KP activity are involved. Firstly, there is a key role in determining the balance of immune system activity between pro- and anti-inflammatory status, a view initiated by the discovery that the first enzyme of the KP—indoleamine-2,3-dioxygenase (IDO)—was induced by the inflammatory protein interferon-γ (IFN-γ) or by bacterial lipopolysaccharides (LPS, ‘endotoxin’) indicating the presence of infection [1]. This link carries implications for understanding the origin and treatment of autoimmune disorders and cancer.

Secondly, studies of neuronal activity in the central nervous system (CNS) revealed that two of the kynurenine metabolites were active—quinolinic acid as a selective agonist at glutamate receptors sensitive to N-methyl-D-aspartate (NMDA), while kynurenic acid was an antagonist (see [2]). Glutamate receptors are now known to be fundamental in synaptic transmission and neuronal plasticity, with the associated development of what is now recognised as a ‘neuroimmune interface’ between the nervous and immune systems [3]. This concept provides a potential mechanism of bidirectional communication between the immune and nervous systems, linking peripheral systemic stress, infection and inflammation with the CNS functions of cognition and behaviour [4,5]. 

Thus, from its humble beginnings as an acidic substance isolated from canine urine and descriptively named ‘kynureninsȁure’ [6], kynurenic acid (kynurenate is the ionised form at physiological pH) has become a key compound to understanding the nervous system, the immune system and their interactions [7,8]. There is strong evidence for a role of the KP in neurodegeneration, potentially involving neuronal over-activation by quinolinic acid and 3-hydroxykynurenine (3-HK), and in psychiatric disorders in which reduced neuronal activity caused by excessive kynurenic acid seems to be involved, especially in the impairment of cognition. With the presence of a glutamate receptor agonist and antagonist in the KP, this qualitatively dual spectrum of activity has led to much interest in the ratio between kynurenic acid and quinolinic acid (the ‘neuroprotective ratio’) in a number of disorders.

The primary objective of this article is to take a sideways look at the field, focusing on outstanding questions, problems of interpretation, areas of confusion and situations in which taking a different perspective might prove valuable in obtaining a more rounded and robust appreciation needed for further experimentation and understanding. This is particularly important as the KP has entered a phase in which selective, targeted compounds are in late stages of development or in clinical trials for medicinal uses in human patients [9]. In many of those situations, kynurenic acid and its sites of action seem likely to be among the more important elements pathologically and therapeutically. It is therefore increasingly important to take account of as many practical issues and considerations as possible.

## 2. Biological Issues

This section aims to examine aspects of data interpretation and the relationship between concentration and activity, targets and outcomes, cause and effect and other factors which should be appreciated more widely in the analysis of kynurenine and kynurenic acid research. Since many reviews on IDO-KP have appeared on specific topics, these will be cited as appropriate and should be consulted for the earlier literature, although key early observations will be cited in many cases. The emphasis will be on more recent notable developments. 

### 2.1. Generation and Movement of Kynurenic Acid 

#### 2.1.1. Cells and Transporters

**Figure 1:**
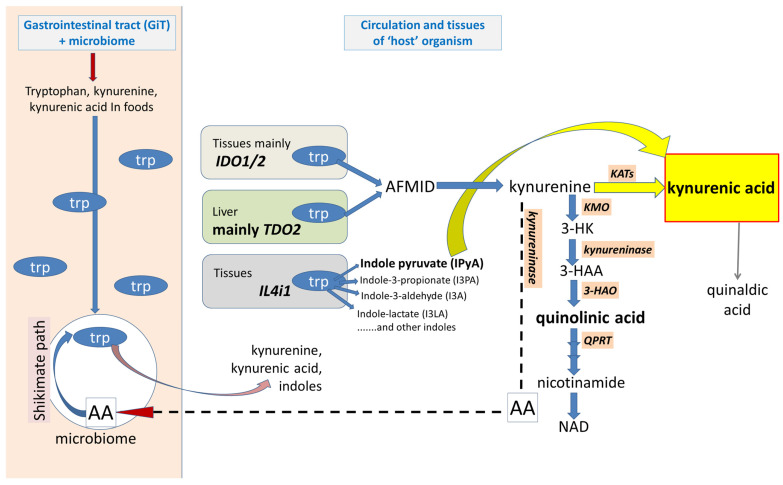
Synthesis and sources of kynurenic acid. A summary of the synthetic pathway for kynurenic acid. The dominant enzymes IDO1 and IDO2 are in the tissues, and TDO in the liver. Tryptophan is present in the GiT and in dietary foods, and is synthesised from anthranilic acid in bacteria. The amino acid and its metabolites in the GiT, including components of the kynurenine pathway, readily enter the host circulation and tissues. The enzyme IL4i1 in tissues metabolises tryptophan directly to simple indole compounds including indolacetate, indole-3-propionate and indole-3-pyruvic acid. The later then spontaneously cyclizes to kynurenic acid. Trp: tryptophan; AFMID: arylformamidase; 3-HK: 3-hydroxykynurenine; 3-HAA: 3-hydroxyanthranilic acid; 3-HAO: 3-hydroxyanthranilic acid oxygenase; QPRT: quinolinate phosphoribosyltransferase; AA, anthranilic acid.

Once synthesised by intracellular kynurenine aminotransferases (KATs) or interleukin-4-induced protein-1 (IL4i1), a proportion of kynurenic acid escapes into the extracellular space (Figure 1) This ‘release’ has been demonstrated in vivo [10,11,12] and in vitro by a variety of cell types [13,14,15,16,17,18]. However, a definitive distinction is not always made between kynurenic acid release after being formed intracellularly, and that which might be generated from extracellular kynurenine by KATs, from tryptophan by IL4i1, or functionally comparable, competent enzymes in the extracellular spaces. No enzyme has absolute selectivity for one substrate, and many enzymes have poor selectivity between several.

The extent to which kynurenic acid release results from a simple passive diffusion process [19] or another calcium-independent route [13,16], rather than an active process, is not always clear. Certainly, many cells, including astrocytes in the CNS, express active transporters for amino acids and kynurenine [20]. The transporters therefore exert a significant, albeit indirect, effect on kynurenic acid levels as the low Km for kynurenine at KAT2 means that kynurenic acid production is dependent on the supply of kynurenine as a substrate. When the human organic anion transporters (OAT)—OAT1 and OAT3—were expressed in Xenopus oocytes, kynurenic acid was transported with a similar efficacy as *p*-aminohippuric acid (*p*AH) and oestrone sulfate, respectively [21,22]. Conversely, kynurenic acid inhibited *p*AH and oestrone transport with IC_50_ values in the low micromolar range and transport by both proteins was inhibited by probenecid. The importance of OATs and of the multidrug resistance associated protein 4 (MRP4) were supported most recently by Ma et al. [23], who showed that pharmacological inhibition or genetic deletion of OAT1 or OAT3 increased serum concentrations of kynurenic acid, while inhibiting MRP4 increased levels in renal cells. Deleting MRP4 from hepatocytes reduced the efflux of kynurenic acid into circulation. A related study demonstrated a three-fold rise in plasma concentrations by probenecid alone or in combination with furosemide, confirming that the OAT transporters were a prominent factor in determining the movement and distribution of kynurenic acid into and out of the circulation and tissues [24]. Similar uncertainties apply to kynurenic acid crossing the blood–brain barrier, where passage is normally measurable, but low [25]. It is likely that a combination of the OAT transporters and LAT-1 will contribute to that movement of kynurenic acid, supplementing diffusional transfer.

The Large Neutral Amino Acid Transporter-1 (LAT-1) (Figure 1) is a major mechanism which carries kynurenine into several types of cell where the amine can activate the intracellular targets discussed below [26,27]. At least part of kynurenine transport is linked with the counter-movement of tryptophan. Importantly, transporter expression is enhanced by interferon-γ [28] or IDO1 [29], thus maintaining intracellular levels of kynurenine at times of increased KP drive. 

**Figure 2:**
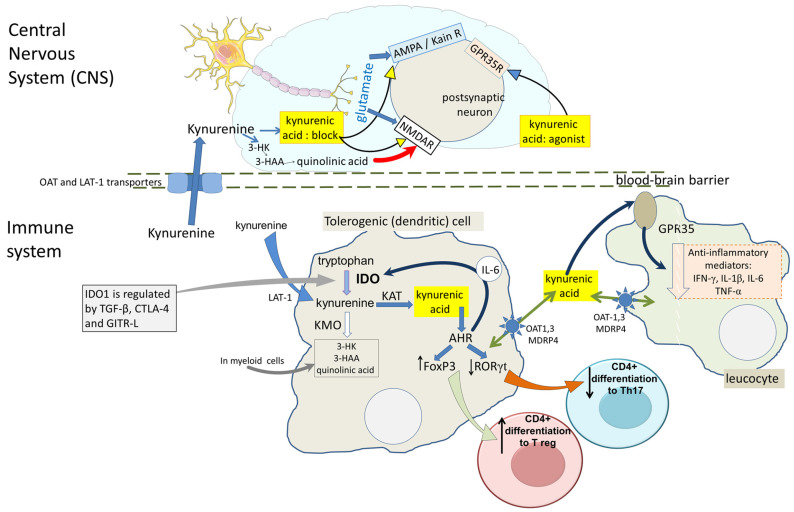
Kynurenic acid: main sites of action. A summary of the major targets responsible for the actions of kynurenic acid is provided here. In the CNS, glutamate—as the dominant excitatory neurotransmitter—acts on ionotropic receptors characterised by their sensitivity to NMDA, AMPA or kainate. Kynurenic acid blocks all three receptors on postsynaptic neurons, but has its greatest effect on NMDA receptors where it blocks the glutamate binding site on the GluN2 subunit, and the (strychnine-resistant) glycine-B co-agonist site located on GluN1. In contrast, kynurenic acid is an agonist at the GPR35 protein, where it has been reported to inhibit neuronal activity and glial function. Kynurenic acid is generated in neurons and glia and also gains entry from the systemic circulation by passive diffusion and active transport across the blood–brain barrier by LAT-1 and OATs. In immune system cells, kynurenine and kynurenic acid are produced by the activation of IDO1/2 or TDO in myeloid cells and tolerogenic dendritic cells. Kynurenic acid then activates AHRs, which promote the differentiation of naïve CD4+ T cells to regulatory T cells (Tregs) via the induced expression of FoxP3, and inhibits differentiation to IL-17-producing cells. The production of kynurenic acid is maintained by a positive feedback cycle via the AHR-induced expression of IL-6 which then induces further IDO1. IDO activity is also maintained and regulated by TGF-β released from activated macrophages, by Cytotoxic Lymphocyte Antigen-4 (CTLA-4), and by the Glucose-Induced TNF Receptor-Related Ligand (GITR-L). The activation of GPR35 in leucocytes inhibits their production of inflammatory mediators such as IFN-γ, IL-1β, IL-6 and TNFα.

It has been argued that, in the CNS, astrocytes are a primary source of kynurenic acid consistent with the presence of KAT2 in these cells [30,31]. The amount of kynurenic acid produced is likely to be higher than in many cell phenotypes since astrocytes lack the downstream enzymes of kynurenine metabolism such as kynurenine-3-mono-oxygenase (KMO) [13,32,33]. Microglia, however, express all components of the KP, including KMO (Figure 2), thus providing the ability to produce the selective endogenous NMDA receptor agonist quinolinic acid [34,35]. Neurons may have this ability since they too have been found to express KMO [30,31]. This cellular variation of enzymatic activity has been the source of much debate since the balance between the production of quinolinic acid and its antagonist kynurenic acid could play a key role in some neurodegenerative disorders [36,37,38] (see below).

An important source of kynurenic acid is the microbial content of the gastrointestinal tract (GiT) (Figure 1). Many bacterial species synthesise tryptophan from anthranilic acid via the shikimate pathway, then metabolise it to kynurenine and kynurenic acid which can gain entry into cells in the intestinal wall and ultimately systemic tissues of the host [39,40,41]. Kynurenic acid has been identified and quantified in a number of foodstuffs which contributes to this pool [42,43]. An important question is that of how much kynurenic acid in the host tissues is produced locally and how much is of bacterial origin. The answer to that would carry substantial implications for understanding the influence of diet and intestinal health on host physiology and pathology, including activity of the CNS [44,45,46]. 

#### 2.1.2. IDO1 Expression

The induced expression of IDO1 generates kynurenine, leading to the formation of kynurenic acid and other downstream metabolites. Expression is most commonly induced by IFN-γ released from antigen-activated Th1 T cells, Natural Killer (NK) cells and CD8+ effector cells. Other inducers are effective, albeit less potent, including IL-6 and Tumour Necrosis Factor-α (TNF-α) which are active themselves to a limited extent, but which are most effective in combination with interferons. There are additional modes of IDO activation which are not dependent on the presence or contribution of interferons, the most important of which is induction by LPS and its activation of Toll-Like Receptors (TLRs), especially TLR4. The pathway involves jun-N-terminal kinases (JNKs) and NFκB activity [47,48,49].

The generation of kynurenic acid by the IDO pathway is raising interest in these alternative routes of activation. A recent study has noted that some serine proteases can activate a very similar pathway to that of LPS, via TLR4, JNK and STATs, leading to the activation of NFκB and its subsequent induced expression of IDO1 and IDO2 [50]. These serine proteases are of considerable clinical importance since they include at least three which have been implicated in tumour formation and cancer progression. These are Prostate Specific Antigen (PSA), high temperature requiring protein A-1 (HtrA1) and the leucocyte membrane protein CD26, which is a dipeptidyl-peptidase-4 enzyme, all of which belong to the class of chymotryptic serine proteases. PSA levels in the blood are now used as the basis of algorithms which, by assessing the changes in its concentration with time, or its levels in relation to other prostate products, can produce a much more robust and reliable pointer for cancer diagnosis and prognosis than simple plasma levels of PSA alone. Since IDO expression promotes immune tolerance and therefore tumour escape, its induction by these serine proteases may contribute to their carcinogenic activity [51]. 

HtrA1, produced by some species of mammals and some bacteria, also induced IDO activity, as did the bacterial enzyme subtilisin, an enzyme frequently used in food processing and cleansing materials [52]. Individual HtrA proteases are important mediators of stress responses. HtrA1 is highly conserved across strains of *H. pylori* and performs crucial intra- and extracellular functions which have led to it being considered as a potential target for anti-bacterial therapy. The leucocyte membrane protein CD26/DPP-4 enzyme is the target of the gliptin family of drugs for the treatment of diabetes and metabolic disorders. 

It will be interesting to assess the contributions of kynurenine and kynurenic acid—generated by the induced IDO—to the biological activity of these enzymes, especially in view of their immuno-suppressive effects which facilitate the immune escape of tumours [53,54,55,56,57,58]. Part of the anti-cancer activity of kynurenic acid may be due to its conversion to quinaldic acid. Although only a minor metabolite of kynurenic acid, quinaldic acid has been demonstrated in human suppressed tumour cell and synoviocyte motility [59,60] and can inhibit the synthesis of insulin [61].

The induced expression of IDO1 by LPS may be an important contributor to the induction of cancers by some bacteria, such as *Helicobacter pylori*, an organism which causes chronic gastritis and is the most common cause of peptic ulcers [62,63]. *H. pylori* induces a high expression of IDO1 which could produce increased tumorigenic immune tolerance.

#### 2.1.3. IDO1 Efflux

Although IDO1 is considered to be an intracellular enzyme, it is possible that a proportion of circulating kynurenic acid arises from IDO1 activity in the bloodstream. Many other large molecules, including proteins such as IDO, can be secreted as a component of extracellular vesicles including exosomes (secreted by most cells), or as a result of cell damage, senescence or death. IDO-containing extracellular vesicles are widely secreted by rapidly proliferating cells such as those of breast cancer [64] and various types of stem cells [65,66,67,68,69,70,71]. As a result, IDO release is part of the rationale for using activated cells, primarily stem cells, as protein carriers in the treatment of severe autoimmune disorders. The underlying assumption is that the secretion of IDO in vesicles would generate kynurenine which would pass into T cells via LAT-1 [72,73]. The cellular uptake of kynurenine, together with its subsequent metabolism to kynurenic acid, could then result in the activation of Aryl Hydrocarbon Receptors (AHRs).

It must be emphasised that the method of quantifying IDO in the blood is problematic. A large number of studies refer to the levels or activity of IDO in plasma or serum based entirely on measurements of the kynurenine/tryptophan ratio (K/T ratio) but this does not provide reliable estimates of the presence or activity of the IDO1 enzyme, as the ratio can reflect changes in the activity of other KP enzymes such as tryptophan-2,3-dioxygenase (TDO), KMO, kynureninase and KAT, as discussed in detail by Badawy and Guillemin [74]. Indeed, the K/T ratio in plasma does not correlate with IDO1 or IDO2 expression in peripheral blood mononuclear cells (PBMCs) [75]. Although there remain uncertainties about extraction procedures and antibody specificity, the quantification is more reliable and meaningful using antibody-based systems such as ELISAs, or chromatographic methods for IDO protein. These techniques have provided unequivocal evidence of IDO as a soluble protein in the serum or plasma [72,73,76,77,78,79,80,81,82,83,84,85,86,87,88,89,90]. 

#### 2.1.4. IDO1-Independent Production

In addition to KP, the enzyme Interleukin-4-induced protein-1 (IL4i1) has been identified as an alternative, albeit indirect, generator of kynurenic acid [91]. As a prominent L-amino acid oxidase, IL4i1 metabolises tryptophan to several indole compounds including indole-3-pyruvic acid (I3PyA) (I3PyA is used here to distinguish indole-3-pyruvic acid from I3PA used for indole-3-propionic acid), which is known to convert spontaneously to kynurenic acid [92,93]. Some emphasis has been placed on the fact that IL4i1 is released from cells as it includes an appropriate signal sequence for protein secretion, but since IDO can also enter the extracellular spaces and blood as noted above, there may be competition with IL4i1 for tryptophan as a substrate. The extent to which that possibility is biologically relevant remains uncertain, since the Km values for IL4i1 oxidation of tryptophan are around 10-fold higher than the Km of IDO1 [30,94,95,96]. Nevertheless, the protein concentration, activity, substrate competition or interaction, modulation, destruction and inhibition are among the factors which could alter their overall and relative biological importance [96,97]. In their study of tryptophan metabolism in healthy subjects and patients with ovarian cancer, Grobben et al. [94] reported that the levels of tryptophan, kynurenine and the K/T ratio were changed in the patient group, whereas there was no change in the level of kynurenic acid. The putative precursor of kynurenic acid generated by IL4i1 was undetectable. This intriguing result was interpreted to suggest that tryptophan metabolism is dominated by dioxygenase activity rather than IL4i1 activity, consistent with the work of Grobben et al. [94]. Although it is necessary to ascertain whether these observations and conclusions have a wide applicability outside the cohorts studied, it could have very significant implications for understanding tryptophan metabolism and its pharmacology.

It is important to recall that Il4i1 can employ several major amino acids as substrates, such as phenylalanine and tyrosine, while IDO1 can act on a wide range of indole-derived compounds including tryptamine and melatonin. In addition, the L-amino acid oxidase- mediated metabolism of amino acids yields α-ketoacids, which could interact with KAT activity, and hydrogen peroxide. These and other possibilities of mutual interference between the KP and other metabolic parameters is an area needing much further research. Efforts to identify selective inhibitors of IL4i1 are already achieving some success [98,99].

The discovery of IL4i1 has moved kynurenic acid closer to centre stage in understanding the importance of tryptophan metabolism in immune system function, as it is the only common product of the IDO and IL4i1 pathways. One of the dominant mediators of immune tolerance is the AHR (see below), for which kynurenic acid is a good agonist [91]. Ramos-Chavez et al. [100] have discussed the apparent presence of alternative, non-KAT-mediated mechanisms for kynurenic acid formation, which may involve IL4i1 and other amino acid oxidases and for which free radical oxygen species are intimately involved [101].

The cellular content of kynurenic acid can be increased by inhibiting KMO, which also reduces levels of the potentially neurotoxic metabolites 3-HK and quinolinic acid, and their synergistic activity [102,103,104]. The endogenous levels of kynurenic acid can increase 10–100-fold, substantially exceeding that of kynurenine [105,106] (Figure 1), with similar increases in plasma and tissue levels observed after deletion of the *KMO* gene [107,108]. Although KMO inhibition may increase the levels of anthranilic acid and its oxidation to quinolinic acid, it is not clear whether this would outweigh the beneficial effects of the raised kynurenic acid. Several KMO inhibitors have been produced, such as Ro61-8048 [109] with others in development [110,111,112].

In addition to neuroprotection, KMO inhibition may prove valuable in the treatment of autoimmune disorders [113], since the increased AHR activation by kynurenine and kynurenic acid will promote the differentiation of naïve CD4+ T cells to anti-inflammatory, regulatory T cells (Tregs) (Figure 2). 

#### 2.1.5. Kynurenic Acid Concentrations

It is difficult to provide a meaningful estimate of how much kynurenine is metabolised to kynurenic acid relative to 3-HK, since this will depend on many factors including enzyme expression, substrate concentrations, time course under consideration and others, and is likely to vary in different tissues. The value of resting levels is addressed below, but KMO inhibition in vivo can elevate kynurenic acid levels by an order of magnitude more than kynurenine [105,106]. This would be consistent with evidence that KAT2 expression in the CNS is an order of magnitude higher than KMO [114], suggesting that under resting conditions the level of kynurenic acid will be maintained at the expense of the quinolinic acid arm of the pathway. A study of oral administration of tryptophan to human subjects revealed an increase in plasma kynurenic acid four-fold more than kynurenine, consistent with the low affinity of KAT for kynurenine producing a rapid transamination [115].

A significant question is that of the kynurenic acid concentrations which are attained in the intercellular spaces and the concentrations reached at its various sites of action in vivo. The variety of analytical methods and conditions under which biological samples are taken, stored and used is sufficiently wide that simple statements of concentrations are very difficult. There are also significant differences between methods based on high performance liquid chromatography and mass spectrometry. There can appear to be a mismatch between levels in the circulation and extracellular medium and the concentrations necessary to act on the proposed receptors or other targets. Most groups have recorded levels of kynurenine in serum around 3 micromols/L [116]. Levels of kynurenic acid are significantly lower, in the range 5–100 nanomols/L, with most reports indicating less than 25 nanomols/L [117]. In contrast, the recognised targets discussed below are said to require significantly higher levels. One of the early studies comparing a wide range of potential ligands reported a Ki of 15 micromols/L at the glycine-B site of the NMDAR [118]. Such apparent disparities are common to many small molecule ligands, including the major amino acids and monoamines, and it is probable that several factors contribute to the apparent discrepancies.

In particular, tissue measurements can be highly variable since the selection, preparation and treatment of tissue samples often differs more between laboratories and experimenters than obtaining blood samples. Generally, tissue levels are much higher than blood levels, with up to 800 nanomols/L for kynurenic acid in human liver and 1–16 micromols/L in porcine colon [119,120]. The level of kynurenic acid in normal human serum is around 30 nanomols/L, while in patients with late-stage kidney disease, serum levels may reach 5 micromols/L [121]. These results were taken to support the concept that kynurenic acid concentrations would be sufficient to activate human AHR in at least some tissues, reaching levels comparable to kynurenine under pathological conditions. Of special relevance here, therefore, is that kynurenic acid may reach levels which would interfere with glycine (and other potential endogenous ligands such as D-serine) binding to gly-B sites on NMDARs, comparable with the value of 15 micromols/L documented by Kessler et al. [118].

#### 2.1.6. Factors Affecting Concentration–Effect Relationships

Among other factors affecting the active concentrations of compounds are the timeframe being examined, the distances between synthesis and action and the kinetics of target activation.

*The time factor*. Most experimental paradigms are relatively brief, time-limited events. Compounds are applied to tissues for periods of minutes or hours, and the concentrations used are those that produce an observable, measurable, statistically significant effect. It would be anticipated that much lower concentrations would activate or block receptors if they were maintained for more than a few hours, perhaps days or, in the case of human patients with a medical condition, possibly several years. In the case of kynurenic acid, this is an especially relevant consideration in view of its mechanism of action on glutamate receptors. As discussed in more detail below, it is a competitive antagonist at glutamate binding sites on ionotropic glutamate receptors for AMPA, kainate or NMDA, but a non-competitive antagonist at the co-agonist glycine site on NMDARs. The lack of any competitive displacement could result in a longer duration, cumulative blockade of glycine than of glutamate. The dual sites of action largely account for the higher potency of kynurenic acid when blocking NMDARs compared with AMPA or kainate receptors, as first demonstrated in the hippocampus in vivo [122]. Our molecular docking data are in the main supportive, with kynurenic acid docking scores in decreasing potencies being NMDA = AMPA, GluA2 > GluA4 > kainate > GluA3 (unpublished observations). Lower concentrations should, for example, suffice to block glutamate sites more effectively after intracerebral microdialysis lasting several hours into a highly restricted volume of a few microliters of tissue. 

*Distance*. The levels of compounds measured in extracellular fluids can only reflect organismal concentrations after substantial dilution from their site of generation. As kynurenic acid effluxes from cells, its concentration at the point of release at the cell surface could easily reach millimolar levels or higher, but since this decreases rapidly with distance (to 1/distance^2^) it is a few tens of microns. It would therefore be expected that the concentrations found could fall to very low levels within blood or CSF would be massively diluted to the levels detectable with current methodology. That they are measurable at all emphasises that the ‘distance factor’ represents only the final concentration after passing through several tissues with different rates of synthesis, release, uptake and metabolism. Any changes in concentration are then difficult to link reliably to the activity in a specific tissue or cell phenotype, especially in an organ as intricately complex as the brain.

*Kinetics*. It is essential to recall the kinetic considerations of kynurenic acid noted above, especially the non-competitive association with the NMDAR glycine site. The measured levels of kynurenic acid may have been present for many months or years, causing not only a chronic inhibition of glutamatergic neurotransmission but also the secondary and subsequent changes that would be induced in other neurons and synapses throughout the CNS. Cognition and its components—awareness, attention, learning, thinking, reasoning—are all subtle aspects of consciousness that require the finest degree of inter-neuronal communication, any or all of which could readily be compromised by interference from long-term, persistently increased levels of kynurenic acid. 

The problem is exacerbated by the fundamental importance of glutamate in CNS neuronal communication. Since all its ionotropic receptors (NMDA, AMPA, kainate) are blocked by kynurenic acid, even a very small reduction in excitability will reduce the activation of subsequent neurons in a sequence, which will then have a reduced effect on the following cell(s). The central role of glutamate thus results in a progressive, entrapped negative feedback, which effectively amplifies a small local inhibition by kynurenic acid into a network-generated functional disturbance apparent at the cognitive and behavioural levels.

*Interactions.* It is probably rare to have only one compound active on one target at any one time. The existence of several potential target actions of kynurenic acid, as outlined below, illustrate the importance of considering its endogenous concentrations. Many studies focus on a single species of receptor, and attempt to dissect the various contributions they make to a particular effect. However, the normal physiological condition of most tissues will involve the presence of multiple receptors being activated around the same time by several ligands. Furthermore, those various sites will tend to be responsive to other ligands, all varying in concentration, duration, kinetics and pharmacological activity, so that the assessment of the role of any one target for one ligand, while being scientifically interesting, may be physiologically or pathologically inappropriate. The precise relative timing and durations of action will need to take into account the time-dependent variations in ligand concentration, the kinetics of association and dissociation from each target and variations in ligand activity and removal by uptake or metabolism. Partial agonists generally exhibit agonist properties at low concentrations, but antagonism at higher levels as the target sites become saturated. In addition, the prolonged exposure of cells to nominally low levels of compounds will usually lead to cumulative adaptations in their targets over a period of weeks, months or years. The complexity of susceptible targets, concentration-dependent actions and compensatory adaptations could result in markedly different effects from those observed in the short term. This may be important, especially in the intestine or tumour microenvironment where the high density of AHR ligands may act synergistically.

While these considerations are relevant to the study of all other biologically active compounds, there is a major need for the testing and examination of interactions between kynurenic acid and other ligands and/or receptors: as with any other substance, kynurenic acid will never be acting alone, but in concert with numerous other compounds generated from nearby cells, exosomes and diffusional movement. Although a concept of fundamental importance in a tissue as complex as the CNS, it rarely receives enough attention.

#### 2.1.7. Kynurenic Acid in the CNS

Special consideration is needed when discussing the CNS, in view of the existence of the blood–brain barrier (Figure 2). Direct comparisons of kynurenic acid levels are difficult as they are often based on different units of measurement, but most estimates of kynurenic acid in the CNS are normally between 2 and 20 nanomoles/L [10,122,123] although resting levels up to 10-fold higher have been observed in human brain tissue [16,42,124,125,126]. The concentrations of kynurenic acid will depend on the identity and activity of its synthesising enzymes. Four kynurenine aminotransferases have been identified to date [127,128]: (1) KAT I [glutamine transaminase K, GTK/cysteine conjugate beta-lyase, CCBL1]; (2) KAT II [aminoadipate aminotransferase (AADAT)]; (3) KAT III (CCBL2); and (4) KAT IV [glutamic-oxaloacetic transaminase, GOT2/mitochondrial aspartate aminotransferase, mASPAT]. The regional and cellular distributions of these are different, with distinct enzyme kinetics and substrate requirements [32,129,130], so their activity will be dependent on general cell metabolism. In the CNS, the most important enzyme, KAT-2, is localised mainly to astrocytes [32] with some in neurons [31,131].

Relatively low concentrations of kynurenic acid have repeatedly been shown to exert clear, significant effects on neuronal activity, cytoprotection and transmitter release. Nanomolar levels reportedly modified the activity-induced release of amino acids and amines [15,132,133,134,135]. Such comparisons, however, should take into account the different levels observed at different ages. Rodents exhibit a 50-fold rise in plasma kynurenic acid over their lifetimes [123,124], with levels rising also in the CNS [136,137,138]. In general, the observed levels would often be sufficient to activate AHR [117]. 

Kynurenic acid levels were clearly identified as being higher in the cerebrospinal fluid (CSF) of patients with schizophrenia (1.67 nanomols/L) compared with healthy subjects (0.97 nanomols/L) [139], results similar to those of Linderholm et al. [140] (2.03 nanomols/L in schizophrenia, 1.36 nanomols/L in controls). Measurements in the prefrontal cerebral cortex (PFC) have recorded comparable levels of around 10 nanomols/L [141], although these cannot be compared directly with most other data as they were expressed as mols/mg protein, which would be expected to yield a higher figure than tissue or CSF measurements. More recent data on twins, in which one had been classified as having schizophrenia, showed that the first affected twin of a pair had kynurenic acid levels in the CSF of 5.6 nanomols/L, compared with the unaffected child (3.1 nanomols/L) [142]. 

In addition to schizophrenia, CNS concentrations of kynurenic acid have been assessed in a variety of disorders, such as Alzheimer’s disease where levels were approximately 52 nanomols/L compared with 19 nanomols/L in controls [143]. These values are clearly higher than usually reported, but surprisingly suggest increased neuroprotection by kynurenic acid rather than the expected reduction. However, a more recent study has confirmed this finding qualitatively, with levels of 3.5 nanomols/L in patients with Alzheimer’s disease and 2.8 nanomols/L in controls [144]. The kynurenic acid/quinolinate ratio was also increased (0.11 vs. 0.08). This counter-intuitive result may suggest that the KP carries two separate implications for understanding Alzheimer’s disease. Over-production of quinolinic acid may contribute to an excessive calcium influx leading to neuronal loss and degeneration, while supranormal levels of kynurenic acid might hinder glutamate-mediated neurotransmission to account for the loss of memory and cognitive confusion.

## 3. Sites of Action

Kynurenic acid has access to target sites located intracellularly and others facing externally. The AHRs are present in the cytoplasm, from where activation by kynurenic acid triggers their translocation to the nucleus. Glutamate (NMDA) receptors and GPR35, however, are located in the cell membrane, responding to the presence of kynurenic acid in the extracellular medium.

### 3.1. Glutamate Receptors

There seem to be several molecular sites at which kynurenic acid may be active physiologically or pathologically. This presents one of the most difficult areas of data interpretation: establishing the identity of these targets and their relative importance. The problem is complicated by many of the issues discussed above, since the normal or abnormal concentrations of kynurenic acid will determine which site or combination of sites will be involved in any particular response. The assessment can only be made with confidence by using selective agonists or antagonists to oppose or mimic the effects of kynurenic acid, recognising that the specificity of some ‘selective’ compounds may be less than commonly stated. The ability of kynurenic acid to block glutamate receptors [2,122,145] has resulted not only in much of the clinical interest described below, but also studies in which kynurenic acid has been used as a valuable tool to screen for the involvement of glutamate and glutamate receptors in studies of other neuroactive compounds as mediators of neuronal projections and pathways within the CNS. Its inhibition of neuronal activation through glutamate receptors composed of kainic acid-sensitive subunits (GluKA1–4) and those activated through AMPA-sensitive subunits (GluA1–4) can account for much of the general suppression of neural activity which may underlie its sedative, depressive and anti-convulsant actions (Figure 2). 

As introduced above, glutamate antagonism by kynurenic acid at NMDA-sensitive receptors involves acting partly at the glutamate binding site on the GluN2 subunit, but also blocking the co-agonist glycine-B (strychnine-resistant) site on GluN1 [146,147,148]. Importantly, the former action is competitive, while the latter is non-competitive, giving kynurenic acid a complex balance of activity dependent on ligand concentrations and the time course over which its activity is being considered. The kinetics of overall binding will have different implications for preventing a rapid response to a high concentration of agonist such as occurs during synaptic transmission and is mediated by AMPA and kainate receptors, rather than the slower, lower intensity activation via NMDA receptors and the dual site of kynurenic acid blockade. In this respect, kynurenic acid could be considered to have some characteristics similar to those of the anti-dementia drug memantine, a suggestion supported by a report that the blockade of glutamate receptors by memantine may be due partly to its ability to enhance kynurenic acid synthesis [149].

This mechanistic difference in the balance of activity mediated by NMDA receptors relative to AMPA and kainate receptors will mean that kynurenic acid enhances the contribution of network plasticity relative to basic, general neural excitability. This ‘filtering’ effect may help to account for the substantial decline in cognitive and intellectual function generated by an NMDA receptor blockade. This discrimination will be further enhanced by the non-competitive nature of antagonism at the Gly-B site. Depending on the relative kinetics of association and dissociation, kynurenic acid is likely to have a more prolonged action at the glycine site since it cannot be displaced by glycine or D-serine even when concentrations rise substantially during periods of intense neuronal activity. While general excitability may recover rapidly as kynurenic acid levels decline, therefore, the inhibition of plasticity may persist even as general excitability is restored. Such a dichotomy of actions could explain the occurrence of purely cognitive dysfunction in the absence of any generalised inhibition and sedation. Cognitive function would be more sensitive and more easily disrupted than general excitability and wakefulness by NMDA receptor inhibitors. Indeed, the associated reduction in excitability via the blockade of AMPA and kainate receptors may be more important in generating symptoms such as depression and sedation.

The blockade of glutamate receptors will have not only direct effects, but also indirect consequences associated with a general reduction in neuronal excitability and plasticity. This will generate downstream changes in neuron and glial populations with changes in the production or release of many other neurotransmitter and neuromodulator compounds, cytokines, peptides, lipids and many others.

### 3.2. G-Protein Coupled Receptor-35 (GPR35)

Wang et al. [150] reported that kynurenic acid acted as an agonist at the orphan receptor GPR35. This protein is able to inhibit neurotransmission via G-proteins G(α-i/o) [151], which might result in a functional synergism with the block of glutamate receptors by kynurenic acid and produce a reduction in neural activity (Figure 2). Such a possibility could be important in a clinical setting since it might imply that a medicament which interfered with only one of these receptor families (glutamate or GPR) might have a disproportionate activity in restoring normal physiological function.

The phosphodiesterase-5/6 inhibitor, zaprinast, was also found to be an agonist at GPR35 [150]. Other ligands have been proposed, such as lysophosphatidic acid and the chemokine CXCL17, but doubts have been raised about these possibilities [152]. A recently described ligand is the 5-hydroxytryptamine (serotonin) metabolite 5-HIAA [153,154] possibly generated by IL4i1, as Nilsson-Todd et al. [155] observed that levels of kynurenic acid were correlated with 5-hydroxyindole-acetic acid (5-HIAA) concentrations. Since both kynurenic acid and 5-HIAA are ligands for GPR35, there may be a possibility of synergism between them. 

The potency of kynurenic acid is comparable with its activity at glutamate receptors, with approximately 100 micromols/L regulating complex formation between GPR35 and β-arrestin [156]. It was more active on calcium movements in cells transfected with GPR35 (human EC50 40 micromols/L) and has been shown to modulate monocyte functions at high nanomolar levels [157]. Data from transfected cells, however, may be misleading since the very high levels of receptor protein expressed on the cell surface tends to increase the apparent sensitivity to a ligand.

The role of GPR35 has received significant support from reports that it is widely distributed in neurons and glia in addition to immune system cells (chiefly monocytes, macrophages and dendritic cells) [158,159,160]. The relative functional activity of kynurenic acid at glutamate receptors and GPR35 remains uncertain, influenced by a variety of factors such as their relative numbers, density, location, susceptibility to up- or down-regulation and others. Part of this variability has been ascribed to the use of artificial expression systems rather than cells in endogenous, physiological conditions. Nevertheless, an immunohistochemical study showed GPR35 expression in several regions of the hippocampus, where its activation suppressed neuronal excitability [160], work later repeated by others [161].

Inflammasomes are important contributors to inflammatory disorders, so their potential control may be useful in the prevention of excessive inflammation. One result of kynurenic acid acting on GPR35 is to suppress activation of the NLRP3 inflammasome, thereby inhibiting caspase-1 and cytokine expression in macrophages [162]. This activity would no doubt be enhanced by the ability of kynurenic acid to inhibit the release of several neurotransmitters and inflammatory mediators from cells, contributing to its reduction of inflammation and chronic pain [150,163,164,165]. This includes inhibiting TNF-α release [164,166] and IL-4 release [167] although the production of some mediators such as IL-6 in MCF7 breast cancer cells and splenocytes may be increased by kynurenic acid [168] indicating qualitative differences between tissues. 

The potential value of increasing endogenous concentrations of kynurenic acid to block GPRs in the GiT has been proposed as a useful potential approach to the treatment of intestinal inflammatory disorders, among which Crohn’s disease and related conditions are a major clinical target [169]. Since GPR35 is expressed to a large extent in the GiT, it is possible that its concentration there, together with the relatively high levels of kynurenic acid in the intestinal wall and contents, may play a significant role in those conditions. This view is consistent with direct evidence linking kynurenine metabolism with intestinal disorders [42,169,170].

An additional role for GPR35, which could also result in a synergistic response with kynurenic acid, is its activation of the AHR [168] discussed in the following section. 

### 3.3. Aryl Hydrocarbon Receptors (AHRs)

The activation of AHR by kynurenic acid induces xenobiotic metabolic enzymes such as CYP1A1, a member of the cytochrome P450 family of enzymes. This contributes to the removal of foreign chemicals such as dioxins. Kynurenine was shown to activate AHRs, thus linking them with the IDO-KP axis [171]. In addition, AHRs promote the further induction of IDO1 and TDO2 expression, initiating a positive feedback production of kynurenine metabolism (Figure 2) [171]. These effects were confirmed and extended by Opitz et al. [116] who recorded an EC50 of 37 micromols/L for kynurenine at AHRs, comparable with the estimated endogenous concentration achieved by stimulating endothelial cells with IFNγ (~40 micromols/L). However, kynurenine may not be the primary ligand at AHR [163,172]. Kynurenic acid is more potent, showing significant activity at 100 nanomols/L on the induction of CYP1A1 mRNA in a hepatic cell line, or 1 micromol/L.on primary human hepatocytes. A human breast cancer cell line (MCF-7) responded to kynurenic acid at a physiologically relevant concentration of 100 nanomols/L with an induced expression of IL-6. Murine cells are less sensitive to kynurenic acid, with an EC25 of 10 micromols/L. Kynurenic acid could displace a directly acting ligand from human AHR, indicating its ability to bind directly to the receptor [168].

The activation of AHRs affects the regulation of T cell differentiation in the treatment of autoimmune disorders and the development of cancers. Some of those actions involve effects on adhesion molecules affecting leucocyte migration and chemotaxis [157]. AHR can modulate skin viability and function [173], of relevance to skin physiology, inflammatory disorders and cancer. The tryptophan photo-oxidation product 6-formylindolo(3,2-b)carbazole (FICZ) affects dermal function at nanomolar concentrations [174].

Since kynurenic acid is much more potent on AHR than kynurenine, the latter may be considered comparable to a pro-drug for the delivery of kynurenic acid to its target sites in the sense that the product has greater biological activity than the precursor. A similar comment could be applied to the generation of 3-HK and thus quinolinic acid. Kynurenine concentrations of 10–100 μM fail to activate the AHR in cell culture systems using P-450-dependent drug metabolism and/or gene expression [168,175]. In the absence of controls on further metabolism in culture systems, kynurenine may often be metabolised to kynurenic acid. Molecular docking data of the interaction with the crystal structure of the human AHR in silico demonstrate the absence of binding of kynurenine (A A-B Badawy and S Dawood, unpublished observation).

AHR activation can up-regulate IDO expression in tolerogenic dendritic cells [116,176,177,178,179,180,181] and in epidermal Langerhans cells [182]. In some cases, the induction of IDO is said to be dependent on AHR [183], rather than being a direct effect. There is also an indirect, autocrine feed-forward circuit which maintains IDO activity via the release of IL-6, which induces IDO via STAT3 (Figure 2) [176]. An important factor in this feedback is AHR inducing IDO1 phosphorylation and Transforming Growth Factor-β1 (TGFβ-1) expression in DCs [184,185]. The promotion and maintenance of IDO by TGFβ-1 underlies the ‘infectious tolerance’ [186] which is a key role of the KP in immune function (Figure 2). 

AHR controls a positive forward loop which promotes IDO1 expression involving reactive oxygen species [187] and can regulate the IDO1-kynurenic acid axis via LAT-1. Expression of this transporter is increased by AHR [188], thus enhancing kynurenine uptake which may be metabolised to kynurenic acid. Conversely, several endogenous L-amino acids—at normal, physiological concentrations—reduce the synthesis of kynurenic acid by inhibiting LAT-1 [189,190]. 

A feedback arrangement via AHR could link the KP with IL4i1. Indole-3-aldehyde (I-3-CHO) is one of the tryptophan metabolites produced by IL4i1 and it activates AHR, thereby inducing IDO and further AHR by kynurenic acid [191]. 

Interestingly, at high micromolar levels, kynurenine and kynurenic acid have been shown to inhibit AHR protein levels despite there being no change in gene expression [192]. AHR can induce or inhibit IDO1 by interfering with the proteasomal degradation of the enzyme. 

### 3.4. Hydroxy-Carboxylic Acid Receptors (HCAR)

The proposal for HCARs as targets for kynurenic acid is a recent development and convincing evidence remains sparse [193]. Nonetheless, the possibility of kynurenic acid binding to HCARs, especially HCAR3, is an intriguing concept as it would expand the potential pharmacological importance of the interactions between kynurenine metabolism and general cell metabolites such as butyrate [194,195]. How kynurenic acid would act via these targets, its relative potency compared with other metabolites and whether there is any significant clinical relevance are likely to prove important questions in the near future. 

### 3.5. Nicotinic Receptors

Although there is little doubt that postsynaptic nicotinic receptors are important in the CNS, physiologically and pathologically, there is good evidence to suggest that many of them are located at presynaptic sites which modulate the release of glutamate [196] and which complicate the interpretation of results in such a complex organ as the brain. The possibility that kynurenic acid can block α7-nicotinic receptors remains very low, with no reported confirmations from independent laboratories of the proposal made in a single publication. There is also a substantial amount of evidence against the idea of nicotinic antagonism [197], although kynurenic acid may block the transmission *process* at nicotinic synapses [198] probably by its block of glutamate receptors causing a depression of neural excitability, or possibly by interfering with the production and release of acetycholine. 

## 4. Emerging Targets and Mechanisms

Having discussed factors that can cause confusion in studies of the KP, and having introduced some of the complications involved which should be considered in future work, it is appropriate to present some of the clinical areas which are showing the greatest promise in terms of the discovery and use of therapeutic agents. The degree of interest is apparent from the website of the World Intellectual Property Organization (WIPO) which on 1 June 2024 listed 4232 patent applications involving the word ‘kynurenic’ (WIPO 2024) [199], used as a reference to its biological activity or that the patent is for analogues or derivatives. Kynurenic acid itself (FS2, FibroStop) has progressed through Phase 1 and Phase 2 clinical trials [200,201]. A good summary of clinical trials based on manipulation of the KP and covering many areas of medicine has been published [9]. 

### 4.1. Is the KP a Cause or an Effect?

When a system such as the KP appears to be involved in such a wide range of biological functions, it raises a fundamental philosophical and practical question. Is the pathway involvement an indication that it is an initiating factor in the cause of the disorders described, or is it perhaps a homeostatic response of the organism to restore normality in the face of disturbance caused by some other cause such as an early tumour, infection, dietary change or stress [202]? At present, there is no clear conclusion. We have proposed a ‘reflex’ scenario which addresses the problem and which could underlie many of the observed activities of the KP [5], but this does not directly establish whether any of those actions are purely physiological or are entrained by pathological triggers. 

Could the KP be a general feature of cell metabolism comparable with the tricarboxylic acid cycle (Krebs cycle) or the adenylate cyclase system? As such, the pathway may act as a compensatory mechanism for basic biochemical and metabolic parameters [203]. This should not, however, undermine the concept of the pathway as a target for manipulating errors in basic metabolism. Any general cellular pathway, including that of kynurenine metabolism, may be a normal component of cell activity, but dysfunction and disease may develop if there is a pathological over- or under-activity in a particular tissue or a specific cell phenotype (such as endothelial or secretory cells) more widely. In addition, for a system such as the KP which generates compounds with specific molecular targets as described above, tissue dysfunction may arise from abnormally excessive or inadequate expression or activity of one of its components or targets. Using therapeutic agents to normalise those abnormalities towards physiologically normal values may be perfectly feasible with little or no disruption of normal cell function. Examples of drug classes to which this view would apply include the phosphodiesterase inhibitors, angiotensin converting enzyme inhibitors, eicosanoid system modulators and β-adrenoceptor blockers. 

A further important consideration is the variability in gene sequences and protein structures. Variants have been recognised of NMDARs, GPR35 and AHR in the tissues of humans and other species, with demonstrated consequences on expression or sensitivity. Similarly, enzymes of the KP exhibit variants, some of which affect activity (Arefayene et al., 2009) [204] and have been linked to specific disorders [205,206]. The most relevant one for kynurenic acid synthesis—KAT2—exists in several variants which have marked effects on the enzyme’s susceptibility to inhibitors [207]. In terms of developing therapeutic agents, it is important that polymorphic varieties of all the major KP enzymes have been identified. Several are already established as contributing to the development, expression or progress of schizophrenia [208,209]. 

### 4.2. CNS Disorders

#### 4.2.1. Neurodegeneration

The neurodegenerative disorders are characterised by a functionally selective neuronal loss such as that seen in the degeneration of nigrostriatal dopaminergic projection pathways in Parkinson’s disease, or the dysfunction of mutant huntingtin expressing cells—especially in the striatum and neocortex—in Huntington’s disease. The general term is also applied to vascular and Alzheimer’s dementias in which there are regions of CNS with neuronal losses less well defined, although in the latter disease cholinergic projections from the basal forebrain to neocortex have long been considered as an early sign. 

There are good reviews of these disorders in general [210,211,212] and more specifically for Parkinson’s disease [88,213], for Huntington’s disease [30,37,104,214,215] and for Alzheimer’s disease [144,216,217,218]. Indeed, some advanced treatments are currently being tested such as the protective effect of cellular micelles carrying kynurenic acid in the treatment of animal models of Parkinson’s disease [219]. This approach is founded in the observation that KAT1 and KAT2 activities were reduced in striatal samples from patients with Huntington’s disease, as were the levels of kynurenic acid [126].

In all these conditions, there is evidence for an inflammatory component. However, since the KP is clearly activated by inflammatory activity, there has been much interest in the possibility that the generation of quinolinic acid would contribute to the neuronal loss by over-activating NMDARs and provoking excitotoxicity. The increased generation of kynurenine followed by its metabolism to quinolinic acid could conceivably contribute to neuronal degeneration in the CNS [141]. This could be countered by the increased production of kynurenic acid and the concept of a kynurenic acid/quinolinic acid balance (K/Q ratio) which could be responsible for maintaining normal neuronal viability. The K/Q factor has become known as the ‘neuroprotective ratio’ since an increase of kynurenic acid or a reduction of quinolinic acid levels would reduce the tendency to an excitotoxic drive. 

A significant amount of evidence has been adduced to support this hypothesis, showing that quinolinic acid levels are higher in patients with dementia, especially Alzheimer’s disease, while other data indicated a lowering of kynurenic acid [217]. A generally protective role for kynurenic acid would be entirely consistent with the recent proposal that the KP functions as a comprehensive organismal protective reflex response to adverse environmental conditions, compounds or threats [5]. These concepts of neurodegeneration and the role of kynurenine metabolites in dementia have been discussed in several detailed reviews and will not be repeated here [211,212,220,221].

However, many of the earlier studies have relied on peripheral, plasma levels of compounds, or on animal models which may not share the same mechanisms, time course or regional selectivity of the human disorders. A recent report of interest, therefore, has been the finding that 311 patients with Alzheimer’s disease had increased levels of kynurenic acid in the CSF, correlating with the presence of tau protein deposits and with cognitive dysfunction [144]. While this result alone does not necessarily contradict the idea of KP involvement in degeneration, it should prompt a re-appraisal of the simple but satisfying concept of the neuroprotective ratio. For example, one possibility is that the roles of quinolinic acid and kynurenic acid should be viewed independently, rather than as a balance, at least in some situations. Thus, increased quinolinic acid could contribute to neuronal loss by overexcitation, but raised kynurenic acid, even at levels which might still be insufficient to block that excitotoxicity, might still be sufficient to depress excitatory transmission and plasticity widely throughout the brain, contributing to the cognitive deficits. This view would be consistent with earlier discussions [222,223]. 

Although raised levels of quinolinic acid acting on NMDARs have usually been considered directly responsible for much of the neuronal hyperexcitability and damage in these conditions, it can also increase glutamate transmission indirectly by stimulating glutamate release from neurons and inhibiting the uptake and removal of glutamate by local astrocytes [224,225]. The ability of kynurenic acid to block all three of the ionotropic glutamate receptors may therefore represent a crucial factor in its neuroprotective properties. In particular, it is possible that it is the relative presence, properties and activation of all three glutamate receptors—perhaps with the metabotropic receptors on a long time scale—which will determine the bar at which the K/Q neuroprotective ratio becomes relevant. 

It remains very possible that the kynurenine metabolites are involved in neurodegeneration of specific cell populations, rather than having a more widespread impact. Huntington’s disease is a good example, where the susceptible cells are expressing a mutant form of the huntingtin protein. Evidence from animal models [107] and correlations between human genetics and KP activity do support a selective role for tryptophan metabolites such as quinolinic acid and kynurenic with the possible involvement of 3-HK [37,226]. A recent report is based on the fact that KMO is localised to the outer membrane of mitochondria, where it forms a complex with a fragment of huntingtin. The functional relevance of this remains unclear, although in cells expressing the mutant form of huntingtin, its complexation with KMO was inhibited [227]. This observation should prove invaluable in clarifying the role of normal and mutant huntingtin, and perhaps in targeting specific aspects of mitochondrial function.

In all these conditions, there are arguments for developing treatments based on interference with the KP. These include recent suggestions that the inhibition of IDO-1 might be a valuable adjunctive treatment to limit the inflammatory aspects of neurodegenerative conditions such as Parkinson’s disease and dementia [88,218]. This would certainly be a rational approach to test for proof of concept although, as implied above, it is difficult to predict what the overall effects would be on the levels of individual KP components and on the impact of changing the ratios between compounds. This is often a vexatious question in view of the range of actions exhibited by kynurenine and its metabolites on neural excitation versus inhibition, redox regulation versus oxidative stress and inhibition versus allosteric potentiation of the various pathway enzymes.

A second group of disorders which are often included in the ‘neurodegenerative’ class are those in which the glial cells, especially oligodendrocytes, are thought to be primarily responsible, such as multiple sclerosis (MS) and amyotrophic lateral sclerosis (ALS, motoneuron disease). These and related conditions, and the roles of KP metabolites, have been the subject of extensive reviews [213,228,229,230,231,232]. 

#### 4.2.2. Neurodevelopmental Disorders: Schizophrenia 

Of the psychiatric disorders, one area which has received most attention and evidence is that of cognitive dysfunction, especially schizophrenia. One of the reasons for this is the appearance of defective memory and executive performance in the offspring of mothers with immune activation [233]. Stressors such as infection and inflammation are important possible candidates for promoting embryonic susceptibility to psychiatric aberrations [234,235,236] and although the mechanisms are far from clear, there is evidence that maternal immune activation may involve the microglia as cells profoundly involved in both the nervous and immune systems [237,238].

As a psychotic condition, the illness is not appreciated by the sufferer and yet it poses serious dangers for the sufferer and others around them often based on abnormal thoughts and beliefs fuelled by auditory or visual hallucinations. For over 50 years, understanding and treatments have centred on dopamine as a major neurotransmitter in striatal projection pathways which govern desires, addiction, emotional performance and social behaviours. Treatments with dopamine receptor antagonists, however, can provide some relief from early stage (or ‘positive’) symptoms, albeit leading to disabling motor problems with chronic administration. In addition, the more severe (‘negative’) symptoms developing in the late stages of the condition do not respond to these treatments and probably involve neuronal damage and death. 

A turning point came with the recognition that a deficit in glutamate receptor activation could be highly relevant to understanding the disorder [239,240]. This was accompanied by proposals that the glycine-B co-agonist site on NMDARs might be a key candidate for therapeutic targeting in the search for improved, more specific and safer drugs [241,242,243]. In addition, there was growing evidence for a role of inflammation, based on the observed changes in inflammatory cytokines, especially IL-6 and other immune system mediators. Kindler et al. [244] have now completed a thorough analysis of some KP components and enzymes, including their mRNA transcripts, in both the plasma and postmortem brain tissue from human patients with schizophrenia. The authors also examined brain volume and cognitive function, together with cytokine correlates of inflammation. Kynurenic acid concentrations (absolute and relative to tryptophan) were increased in the PFC of patients with high levels of inflammatory cytokines. From the overall pattern of results, it was suggested that this was consistent with inflammatory triggers which induced tryptophan metabolism (TDO) and kynurenic acid synthesis (KAT2), leading to the characteristically impaired deficits in attention, the associated cognitive dysfunction and a loss of PFC volume. It would be of interest to know why TDO is activated, and whether there are parallel changes in IDO1-mRNA, the primary target of proinflammatory cytokines. 

It seems increasingly likely that many of the abnormalities seen in the schizophrenic CNS may result from factors acting during the early stages of embryonic development, but persisting into adolescence and adulthood. These can be summarised as maternal stress caused by environmental factors, dietary changes, infections or general inflammatory status. A role for kynurenine metabolites was implied by using the KMO inhibitor Ro61-8048, administered to mice during gestation. The treatments produced substantially raised levels of kynurenic acid (10–100-fold) in the maternal and embryonic tissues [105,106], and resulted in alterations in hippocampal neuron spine type and density, hippocampal electrophysiology and synaptic plasticity, with modified histochemical distribution and density of neuroactive compounds [105,106,245,246]. Later work expanded these observations by showing behavioural changes after the chronic administration of kynurenine to the pregnant dams [247,248,249] which inhibited spatial learning performance. 

An important consequence of these studies has been the realisation that endogenous levels of kynurenic acid are inversely correlated with cognitive performance, an enhanced ability being induced by lowering the concentration using KAT inhibitors or gene deletion. Consistent with this from a clinical perspective, kynurenic acid levels are increased in disorders characterised by cognitive dysfunction, especially schizophrenia [4,108,140,250,251] and related conditions. The induced behavioural changes can affect very restricted aspects of performance. Increased levels of kynurenic acid, for example, inhibited fear discrimination (using an auditory footshock paradigm) induced by several forms of stress [252]. 

A study on the subtleties of cognitive activity revealed that some aspects of associative memory—but not recognition memory—were selectively and highly correlated with the ratio of quinolinic acid to kynurenic acid concentrations in the plasma [253]. While this result is of great interest, concerns about the relevance of blood measurements restrict confidence in the inferences drawn from it. Nonetheless, it suggests that it may be possible to identify those facets of cognition which are most susceptible to damage or protection by alterations in the KP. In a recent meta-analysis, it was concluded that there was evidence for an association between KP activity and schizophrenia symptoms (see [254]), especially the disturbance of cognitive performance [255].

There is an increasing number of examples of intimate relationships between the KP and immune system mediators and modulators [3]. It has been shown that several of these, including IL-1β and IL-6, accumulate in bipolar depressive disorder and schizophrenia, with the ability to activate the KP and increase levels of kynurenic acid [256]. Other routes to activation are likely to be relevant, including the indirect activation of TDO2 via glial caspase activation, and contributions from other mediators including nexin 7 (SNX7) [108,257]. The behavioural and cognitive changes are consistent with earlier work on the effects of kynurenic acid on the firing rates of midbrain dopaminergic neurons [258], neurons known to be intimately involved in social interactions, addiction and reward behaviours. 

In some of these studies, the use of KMO inhibition or kynurenine do not allow a judgement on whether the causative chemical is kynurenine, kynurenic acid or another downstream or indirect product. One instance where this question has been addressed is in the study of memory performance following the prenatal (in utero) administration of kynurenine [249]. Here, it was shown that the usual memory deficits were prevented if the pregnant animals were treated with a systemically active novel inhibitor of KAT-2, confirming a significant contribution, at least, of kynurenic acid. As with any in vivo study, it is important to remember that blood concentrations of compounds may not reliably mirror or predict the levels reached in the CSF or CNS parenchyma [258].

Other approaches are being pursued to assess the general applicability of these various ideas on kynurenine metabolites and cerebral, behavioural activity. One example is predicated on the established human phenomenon that the chronic recreational use of cannabinoids in adolescence carries a strong probability of developing schizophrenic symptoms in later life. It has now been shown that the concentration of kynurenic acid is increased in the PFC of rats treated chronically with d9-tetrahydrocannabinol, a finding consistent with the link between schizophrenia and raised kynurenic acid concentration [259,260].

A different approach is centred on the effects of changing dietary compounds to manipulate KP activity. A recent assessment of plasma concentrations in healthy humans and schizophrenic patients revealed that tryptophan administration increased the levels of kynurenic acid more than six-fold compared with the increase in kynurenine levels [31]. However, these relative levels of increase were similar to those in controls, which may not be surprising given the difference in levels of kynurenine and kynurenic acid (μM vs. nM). Because the tryptophan dose used (6 g) causes maximum activation of TDO in normal subjects [115], it is possible that the effect of KAT up-regulation in schizophrenia might have been masked by contributions from TDO induction and flux of kynurenine down the KP. 

The finding by Sathyasaikumar et al. [31] may have significant implications for KP activity under chronically maintained conditions of tryptophan deficiency or excessive intake. The question may be especially relevant during pregnancy and early postnatal development, when it is known that changing activity in the pathway produces the marked biochemical, structural, electrophysiological and behavioural abnormalities noted above.

Overall, the concept that a blockade of glutamate receptors is a key factor in the regulation of cognition and behaviour via its influence on cell activity and modulator release is now well established and has been discussed with respect to animal models and clinical examples of psychiatric dysfunction [261]. Although discussion is often centred around NMDA receptors, it should be re-emphasised that the block of AMPA and kainate receptors by kynurenic acid will also be relevant, both as a result of their role in neuronal excitability, and also since they will affect the basal state of cells upon which the voltage- and activity-dependent effects of NMDA receptors are dependent.

#### 4.2.3. Depression

Some of the factors discussed above are probably also relevant to Major Depressive Disorder (MDD) and its treatment, since early phases are generally amenable to pharmacological agents, whereas the development of drug resistance has been linked with a loss of regional cerebral volume [262,263]. 

Since 5-hydroxy-tryptamine (5-HT, serotonin) was first discovered in the CNS almost a century ago, its suspected relationship to depressive disorders has become widely accepted, despite the dearth of convincing positive evidence to the contrary and some evidence opposing the idea in animals [264] and human subjects [265]. Indeed, some studies have concluded that psychological counselling is more effective than Selective Serotonin Reuptake Inhibitors (SSRIs) [266]). An increasing volume of data has therefore provoked a shift in mechanistic emphasis away from ST, to one in which the KP appears to dominate the explanatory landscape [267] probably related to parallel changes in inflammatory status [268]. It may be relevant that a metabolite of ST, N-acetyl-5HT, has been shown to positively modulate IDO activity, presenting a clear link between 5HT metabolism and the KP which had not been recognised [269]. 

The neuronal loss which has been described in depression may be associated with glutamate receptor-mediated excitotoxicity [270], especially in bipolar disorder (BD) [211]. However, an MRI investigation of cerebral microstructure in patients with unipolar or bipolar depression [271] revealed that plasma levels of kynurenine were higher in bipolar patients and the K/T ratio was positively correlated with IL-1β and TNF, but negatively with IL-2 and IL-9. The high K/T ratio was also associated with lower white matter anisotropy, leading to the conclusion that the K/T ratio and inflammatory cytokines might be causally linked to the structural changes in bipolar disorder but not MDD. This would be consistent with mice lacking KMO, in which kynurenine and kynurenic acid levels are elevated in association with the development of depression symptoms which responded to conventional antidepressant agents [272]. Similar conclusions were drawn in an examination of KMO and depression in mice, in which depletion of the enzyme prevented the development of depressive behaviours following the administration of LPS [273] 

Using plasma concentrations, a reduced K/Q ratio was described in patients with active MDD or BD and those in remission [262,263]. The study does reduce the possibility that changes in the KP are secondary to the symptoms of depression, such as anhedonia and reduced motor activity. Similar results of reduced kynurenic acid or of the ‘neuroprotective ratio’ K/Q were obtained in related studies [274,275] including a meta-analysis of 101 studies, embracing almost 11,000 patients [276]. The K/Q ratio has been correlated with the amygdala volume, supporting its relevance to neuronal loss which increases as the disorder progresses [262,263]. Furthermore, the link with underlying inflammation in depressed patients is supported by finding that both the K/Q ratio and levels of the inflammatory marker C-Reactive Protein were independently correlated with measures of white matter integrity [277]. Of course, analysis can still suffer from the limitation that plasma levels of kynurenine and its metabolites may not reflect levels in the CSF, which in turn do not necessarily reflect the concentrations present at receptors on the surface of individual cells.

Since any hypothesis is strengthened by the demonstration of its relevance from an opposing viewpoint, it is important to note that increasing the activation of NMDAR using a novel positive modulator was able to prevent or reverse depressive behaviour in mice [278]. Furthermore, it was possible to define the precise site of action of the drug as activating GluN2B-subunit-containing NMDARs on excitatory neurons in the PFC, a result entirely consistent with the arguments of glutamate receptor involvement.

Apart from this possible link between neuronal damage and disease severity, the therapeutic relevance of modulating the KP remains a viable option in view of molecular docking data in silico demonstrating the specific binding of a range of antidepressant drugs to the crystal structure of the human TDO [279] and their failure to bind to IDO1 [280]. The latter authors [280,281] suggested that, whereas TDO is targeted by antidepressants, drugs among these with anti-inflammatory properties are likely to prevent IDO1 induction by lowering proinflammatory cytokine levels. Future studies of depressive illness should consider the potential role of TDO.

#### 4.2.4. The Need for Drug Selectivity

The proposed shift of KP metabolism away from kynurenic acid in conditions such as schizophrenia and major depression may encourage glutamate-induced toxicity to develop in their chronic stages, partly due to compensatory glutamate release or receptor up-regulation. Similar concerns may apply to the use of non-selective KAT inhibitors such as amino-oxyacetic acid which has been associated with neuronal damage [282,283,284]. However, the shift in metabolism raises the risk of increasing the generation of downstream metabolites such as 3-HK, 3-HAA and quinolinic acid which themselves are cytotoxic by promoting oxidative stress and activating NMDARs, respectively. Quinolinic acid and 3-HK can be synergistic in the production of excitotoxicity [102]. Manipulation of this balance of pathway products for the treatment of neurodegenerative disorders will therefore require consideration of the potential secondary and delayed effects of influencing the “neuroprotective ratio”. 

Naturally, the development of any analogues or derivatives of kynurenic acid as therapeutic agents should aim to maximise the target activity while minimising potential sites of adverse events mediated by other target sites. Since the relative expression, activity or sensitivity of target sites often varies in different disorders, this may imply that the amount of therapeutic benefit of compounds will be determined by the relative importance of the targets and their respective abnormalities, as with many other drug classes.

### 4.3. Peripheral Tissues

#### 4.3.1. Cytoprotection: Cytoskeletal Modulation and Wound Healing

One facet of kynurenic acid biology which has received less attention than it might merit is that of wound repair. On co-cultured neuron and glia, quinolinic acid produces a substantial disorganisation of the cell cytoskeleton [285]. Kynurenic acid was able to prevent that disruption in the population of astrocytes, including the loss of connexin-43 and other gap junction proteins, and with a reduction in the degree of oxidative stress. However, these effects of quinolinic did not appear to be mediated through the activation of NMDA receptors, as they were not prevented by 2-amino-5-phosphono-penatanoic acid or dizocilpine (MK-801). Kynurenic acid also prevented the increase in expression of the microglial activation marker Iba1 (ionized calcium-binding adapter molecule-1). The authors concluded not only that these deleterious actions of quinolinic acid may not have involved NMDARs, but also that inhibition of those effects implied that kynurenic acid may also have protective effects independently of NMDARs. However, it is important to note that quinolinic acid is known to be a redox active molecule [286,287] which could explain its NMDAR independent effects, a conclusion which may apply to some effects of quinolinic acid on neurite growth in neurons [287]. As a corollary, it is quite possible that the correction of those toxic effects by kynurenic acid could have been mediated via NMDARs acting in a functionally opposite manner rather than competing directly for the same receptors. Other possible explanations, such as the involvement of AMPA or kainate receptors for glutamate, or actions on GPR35, need to be considered

Viewing kynurenic acid as a cytoprotective compound also applies to its promotion of wound repair. In the aftermath of many tissue wounds, the closure and healing processes can be slowed by the associated inflammatory response and infiltration of activated immune cells. In the presence of kynurenine, the expression of many such mediators is inhibited, with a change in the ratios of cell types. CD3+ cells were particularly suppressed, and the overall rate of healing was increased [288]. There may be a role for the anti-inflammatory cytokine IL-10 in this protection, since kynurenine induces the expression of its receptor, IL-10R [170] by inducing an AHR response element on the appropriate promoter. 

While the use of kynurenine leaves open the question of whether it or its metabolites, kynurenic acid, were responsible for these actions, the latter is much more likely. In a study of Type-II collagen accumulation in fibroblasts, the recovery and scarring from damage was mediated by kynurenine or kynurenic acid and their induced expression of Matrix Metalloproteinases 1/2 [289]. After studying the effects of kynurenine and its metabolites on stem cells, it was concluded that the compounds regulate many aspects of early cell development and differentiation [290]. The precise contribution of kynurenines is varied and depends on the specific cell phenotype being considered, their developmental stage and inflammatory status, the latter conclusion reflecting the potent modulation of tryptophan catabolism by immune system mediators.

Although the effect of changing concentrations in a clinical setting is more difficult to interpret, a study of patients undergoing surgery for colorectal cancer proved instructive. After major surgery for the condition, the sequelae can be severe, including a variety of metabolic changes including physical difficulties. In an extensive study covering a wide range of compounds, it was observed that the most statistically significant correlate of those sequelae was the kynurenine/tryptophan ratio. Furthermore, using hydrogels as a vehicle for kynurenic acid, it has been shown to suppress the inflammation associated with complex perforator flaps during surgery. Kynurenic acid increased neovascularisation and the proliferation and migratory vigour of the endothelial cells examined [291]. The importance of this was reflected in increased patient survival after surgery. 

Recent studies have emphasised the substantial overlap between the cytotoxic and inflammatory factors noted above, with widespread agreement that they may co-exist in some CNS disorders and act primarily in the development and recovery of tissues, especially in the CNS. Thus, having established that cultured glial cells responded to IFNγ by up-regulating IDO expression and producing increased amounts of kynurenic acid and quinolinic acid, O’Reilly et al. [292] transferred the culture medium to primary cultures of neurons. Incubating these neurons with quinolinic acid (1 micromol/L) or the IFNγ-conditioned media inhibited the development of neuronal complexity, with fewer neurites and synaptic markers. Kynurenic acid enhanced neurite growth and complexity and prevented the decline in complexity produced by quinolinic acid or conditioned medium. The results represent a strong consolidation of the concept of kynurenic acid as a physiologically and clinically relevant cytoprotective compound. The results are entirely complementary to the effects of quinolinic acid on neurite growth which are mediated by oxidative stress [287].

Some recent evidence suggests that direct intracerebral administration of kynurenic acid can significantly alter the expression of enzymes regulating redox status, with reduced levels of superoxide dismutase and catalase mRNA in the hypothalamic and hippocampal regions of [293]. Enzyme activity was increased, however, of those enzymes and of glutathione peroxidase. The results show that kynurenic acid can influence the level of oxidative stress in some areas of sheep brain, by affecting those enzymes, a property which may contribute to its cytoprotective activity.

Related results have been obtained from an in vitro study on human brain tissue [294]. A population of radial glial cell progenitors was used, harvested from the cerebral cortex of embryos at mid-gestation (16–19 weeks). The cultured cells developed into a mixture of those glial cells, with neurons and astrocytes. The addition of kynurenic acid inhibited proliferation rate and increased the ratio of astrocytes to neurons. There was also less differentiation to GABAergic neurons. These effects were seen at submicromolar concentrations of kynurenic acid (EC50 of 10 nanomols/L to produce cell death and 50 nanomols/L for reduced proliferation), and appeared to be mediated through the blockade of NMDARs and not nicotinic receptors. The authors reported changed expression of a range of cell markers and properties, including evidence for astrocyte activation as indicated by the increased level of Glial Fibrillary Acidic Protein.

It is possible that there is a more specific relevance of kynurenic acid in discussions of neurodegeneration, that of a role in neurogenesis. One study has claimed that KAT2 is expressed to a high degree in regions of the CNS exhibiting high levels of neurogenesis [295]. Although a role of kynurenic acid in this process would be of great interest, it will be important to study the mechanisms involved and their cellular consequences. Are all components of the KP also affected? What drives KAT activity in these cells, or is it secondary to other facets of the cell cycle and proliferation? Is there any relationship between KAT expression and cell activity, with kynurenic acid perhaps generated to protect cells against locally high levels of glutamate or quinolinic acid? This topic is intimately related to other aspects of kynurenic acid as a factor in tissue repair and regeneration, and could herald a means of promoting functional recovery in the CNS after traumatic or stroke-induced damage.

A different example of the cytoprotective effect of kynurenic acid is in its inhibition of cell death induced by ischaemia. The activation of GPR35 by kynurenic acid is followed by movement of the complex to the mitochondrial outer membrane where it binds to ATP synthase inhibitory factor. This induces dimerization of the enzyme, inhibiting its metabolism of ATP. The increasing level of intracellular ATP delays and reduces cell damage and death triggered by ischaemia [296].

#### 4.3.2. Anti-Inflammatory Properties

Although many of the actions of kynurenic acid described above have direct or indirect anti-inflammatory effects, several additional points deserve mention, one of which is the regulation of inflammatory mediator release. Kynurenic acid can suppress glutamate release from several cell types and reduce the release of inflammatory cytokines such as IL-1β, IL-6 and TNF-α from activated immune system cells, monocytes and microglia [166]. This is probably a major factor in its prevention of severe inflammatory damage or death due to immune system activators such as LPS.

These effects may have been the result of kynurenine conversion to kynurenic acid, a possibility enhanced partly by the production of similar results with the KMO inhibitor Ro61-8048. It is well established that this compound increases kynurenine and kynurenic acid levels in tissues as noted above. However, caution is required in view of a curious distinction reported by Malaczewska et al. [297] in which, using rodent splenocytes in vitro or ex vivo, kynurenic acid was said to suppress the expression of inflammatory mediators when induced by LPS. Using non-stimulated, quiescent cells, however, kynurenic acid increased the release of IL-1β, IL-6 and TNF. This dichotomy of action merits a closer examination, especially with respect to whether the active compound is kynurenine or kynurenic acid, and the identity of the receptor (or receptors) involved. Kynurenic acid also enhances the expression of TNF-stimulated gene-6, a potent inhibitor of immune cell activity and of stem cells [298,299], thus contributing to its overall anti-inflammatory and cytoprotective effects in ulcerative colitis [300] and other disorders [301].

It is especially interesting that kynurenic acid may exert a central role in inflammatory bowel disease. The pathogenesis is considered to be from inflammation, auto-inflammation and auto-antibodies altering the mucus layer and enhancing intestinal permeability which sustains the vicious cycle of further mucosal irritation [302] and may be relevant to a number of CNS disorders [303]. It has been reported that lymphocytes from affected patients exhibit increased expression of the KAT enzymes, generating supranormal levels of kynurenic acid from kynurenine [304]. This may represent a protective reaction to the disease, possibly via GPR35 or AHR activation, in view of the anti-inflammatory and cytoprotective effects of kynurenic acid note above.

### 4.4. Dietary and Metabolic Factors

There is an interface between the routes of kynurenic acid synthesis and general cell metabolism via the production of pyruvate and keto-acids acting on or produced by the KATs. It is not clear how the production of kynurenic acid from kynurenine, its generation from I3PyA and the effects of pyruvate on the Krebs’ cycle interact. It is a question of some importance since pyruvate has a range of actions on cell proliferation, growth, oxidative metabolism and tumour initiation, giving it the potential ability to influence fundamental aspects of major disease development, which may be indirectly affected by activity along the KP. The problem has been made more apposite with the proposal that kynurenic acid can act on those metabolic pathways to alter energy production and utilisation, possibly via GPR35 and AMP kinase activation [305,306].

Kynurenic acid production may be affected by anything which modifies activity of the four known KATs, although KAT-2 is believed to have the greatest relevance in most tissues. KAT-2 can be inhibited by oestrogens [307]. The metabolic product α-amino-adipate also inhibits KAT activity [308], an action which contributes to its gliotoxicity by reducing kynurenic acid’s cytoprotection. The balance between α-amino-adipate and other keto-acids such as pyruvate, which can be substrates for the KATs, could affect the production of kynurenic acid and its accumulation in disorders such as schizophrenia (see below) [309]. 

Dietary modifications could be used to alter KP metabolism to correct metabolic abnormalities, even to the extent that they could be employed as adjuncts to therapeutic treatments. The frequency of seizures, for example, is known to be modified by ketone bodies whose production can be enhanced by a ‘ketogenic diet’ consisting of increased carbohydrate and reduced lipid intake. Some of those ketone compounds have now been shown to affect the KP, reducing circulating kynurenine concentrations in addition to several other general metabolites [310,311]. Simple dietary manipulations could, therefore, provide a useful treatment, or adjunctive procedure, in patients with epilepsy.

Conversely, it has been shown that a ketogenic diet, generating ketone bodies such as acetoacetic acid and β-OH butyric acid, raised kynurenic acid concentration in tissues and protecting retinal ganglion cells against glutamate or NMDA-induced injury [312,313]. This protection may extend to compensating for a loss of retinal kynurenic acid in age-related degenerative disorders of vision since kynurenic acid has been linked to an age-related retinal dysfunction in DBA/2 mice with ocular hypertension [314]. A loss of KAT activity and kynurenic acid concentrations was confirmed in the retina. It has been suggested that β-OH butyrate might act by inducing KAT1 and 2 expression [315,316] and that it might be part of a cerebral protective system against hypoglycaemia.

There are potentially many other methods by which basic cell metabolites could regulate or interfere with the KP and its actions. An example is the observation that butyrate increases the production of 5HIAA. As noted above, this is a positive allosteric agonist for GPR35 and could modulate the activity of kynurenic acid at that site [317]. The autonomic nervous system has a significant effect on kynurenic acid generation. Agonist ligands acting on α- or β-adrenoceptors promote kynurenic acid synthesis in neurons and glia in the CNS, as do cyclic AMP and stable analogues acting via the protein kinase A pathways [318].

#### 4.4.1. Vitamins

Several key enzymes of the KP, especially KAT2 and kynureninase, require pyridoxal phosphate (vitamin B6) as their main co-factor [319]. KMO is an NADPH-dependent enzyme which requires riboflavin (vitamin B2) as a precursor [320], and the need for KMO in de novo synthesis of NAD provides an intriguing feedback circuit. However, since riboflavin is rapidly metabolised in activated immune system cells, KMO may be less active, resulting in increased kynurenine and kynurenic acid levels [321]. This could contribute to the psychological correlates and delirium associated with serious infections and sepsis in view of the proposed role of kynurenic acid in schizophrenia and cognitive dysfunction (see below).

The functional importance of B vitamins in the human KP in relation to the immune system has been confirmed directly [322,323,324]. Cognitive deficits caused by poor nutrition may be caused by these changes in KP enzymes requiring vitamin B, and prevented by monitoring and supplementing vitamins. Nicotinamide (vitamin B3) may improve mitochondrial activity to a degree which alleviates some symptoms of Parkinson’s [325].

#### 4.4.2. Diabetes and Pancreatic Function

The protective aspect of kynurenic acid has been considered relevant to its activity in diabetes. The first suggestion for any involvement of the KP concluded that xanthurenic acid might be the most important element, especially since it binds to insulin molecules and inhibits their enzyme activity without affecting their immunogenicity [326]. The issue has re-appeared with evidence for a role of IDO and the KP in Type-1 diabetes, where it would be expected to contribute to the immune tolerance required to prevent β-islet cell destruction [113], mainly as a result of its presence in the small population of plasmacytoid DCs. The KP is also protective against Type-2 diabetes although the mechanism is not yet clear, with potential utility in treatment of the disorder [327,328,329,330]. The activity of some hypoglycaemic therapies has been associated with an inhibition of kynurenic acid production [331], while the administration of kynurenic acid has been reported to hinder the development of diabetes [306].

It is of interest that kynurenic acid can be metabolised to quinaldic acid, which can inhibit the synthesis of insulin [61]. The dual strike of this action and the xanthurenate ligation of insulin could present a synergistic challenge that might contribute to the aetiology of diabetes.

Pancreatitis is one of the most intractable and frequently fatal disorders. Disorders of the pancreas usually involve a high degree of inflammatory activity, partly stemming from the fact that when its cells are compromised in any way, they are more likely to expose the surrounding tissue to the various potent proteases, lipases, and other enzymes which they normally secrete into the GiT. Furthermore, maintained inflammation can lead to pancreatic cancer. Following earlier work on the role of kynurenines in pancreatic function, it was found that the balance between cytotoxic quinolinic acid and its protective antagonist kynurenic acid was a significant factor in cellular [332]. Therefore, as noted above, the strategy of increasing the balance of kynurenic acid to quinolinic acid was attempted using inhibitors of KMO. It has also been shown that KMO regulates inflammation during critical illness and recovery in experimental acute pancreatitis [333]. KMO primed innate immune and inflammatory gene transcription via 3-HK production, which was synergistic with IL-1β in causing cell apoptosis. Inhibiting KMO reduced the fatal inflammation in pancreatitis. Together, these results clearly support the view that KMO inhibitors are potentially valuable inhibitors of pancreatic inflammation and, thus, of related cases of cancer. Indeed, although kynurenine metabolism is becoming established as a possible marker of diabetes and contributor to the aetiology, it may be a significant ‘checkpoint’ in progress of the disorder [330]. 

The success of this approach has been demonstrated by the recent launch of Kynos Therapeutics, an Edinburgh-based company devoted to the application of KMO inhibition in the therapeutic management of pancreatitis [110,334,335]. It would be surprising if this did not provide a significant test situation, upon which KMO inhibition could be extended to other conditions. The most obvious one is probably Huntington’s disease in the light of the work discussed above, but there are other conditions where lowering quinolinic acid and raising kynurenic acid concentrations could also bring major benefit. It could, for example, be a novel strategy in the treatment of strokes and post-surgical cognitive dysfunction, in both of which correcting the balance between kynurenic acid and quinolinic acid would bring significant promise.

### 4.5. Kynurenic Acid and Cancer

Although inflammation seems to be critically involved in the development of some tumours, a role for the KP in that development and as a therapeutic target has received much attention and will not be addressed in detail here (see [53]). A clinical trial of the IDO1 inhibitor epacadostat in the treatment of melanoma, however, with or without pembrolizumab, an inhibitor of Programmed Death-1 (PD-1), failed to produce a significant reduction of tumour development [336]. This has required some re-thinking of the specific roles of downstream kynurenine metabolites and the continuing development of compounds inhibiting IDO2 or TDO2 in addition to, or instead of, IDO1. Inhibitors of kynureninase and KMO are also being explored [337] and a number of target combinations and sources are under investigation [338,339,340]. 

Levels of kynurenic acid are frequently elevated in sera, bone marrow, intestinal mucosal material and tumours of patients with various cancer types [39,341]. Targeting KAT I or KAT II can limit activation of the AHR by kynurenic acid and its consequences, including involvement of the immune check point IL-4I1. However, KAT1 is expressed in a much larger proportion of tumour cell types than KMO and kynureninase [342,343]. This results in a low level of competition for kynurenine by KMO and kynureninase. In many cases this increases the efficacy of KAT which has a very low affinity for [7]. In all these considerations, it is proving essential to take into account the role of diet and microbially derived indole derivatives, some of which have a marked influence in reducing the efficacy of checkpoint inhibitor therapy against cancer [344].

## 5. Conclusions

One objective of this essay has been to focus on questions and problems surrounding the pharmacology and clinical relevance of kynurenic acid which have not often been discussed or analysed in depth. The article is partly designed to bring these questions more into the open, partly to increase awareness that they are problems which often occur in the experimental sciences, partly to emphasise the need to understand them and, where possible, to add to the present level of understanding.

The second objective is to note the breadth of the clinical relevance of the tryptophan research area, but then to highlight fields of medicine in which the amount of data is sufficiently great, and the drive for understanding and treatment is so urgent, that they are likely to emerge as the harbingers of a new era of clinical pharmacology. Of the many patent applications on ‘kynurenic’ referred to above, it would be surprising if none were turned into valuable therapeutics. Kynurenic acid itself, in a topical preparation as FS2 (FibroStop) has proved clinically acceptable in Phase 1 and Phase 2 trials against scarring and psoriasis, with other indications under consideration [200,201,345]. Inhibitors of KAT2, which reduce kynurenic acid production, are among the most promising candidates in neuroscience for the treatment of schizophrenia and schizoid disorders, while inhibitors of KMO which increase kynurenic acid levels are likely to be of value in regulating the immune system. KMO regulation may greatly improve the prognosis in Huntington’s disease, and its inhibition may revolutionise the treatment of pancreatitis and, potentially, pancreatic cancer.

As research proceeds to more practical levels, it is hoped that the considerations presented here will promote the generation of data with greater validity and reproducibility, and that they will facilitate the translation of robust experimental work more rapidly and reliably into compounds for human therapy.

## Abbreviations

AHR	Aryl Hydrocarbon Receptor
AMPA	α-amino-3-hydroxy-5-methyl-4-isoxazolepropionic acid
BD	Bipolar depressive disorder
CD26	Cluster of Differentiation marker 26, and a dipeptidyl-peptidase-4 enzyme
CNS	Central nervous system
CSF	Cerebrospinal fluid
CTLA-4	Cytotoxic Lymphocyte Antigen-4 (CTLA-4)
cyp 1A1	Cytochrome P450, family 1 metabolic enzyme
GiT	Gastrointestinal tract
GITR-L	Glucose-Induced TNF Receptor-Related Ligand
GPR35	G-Protein Coupled Receptor-3
HCAR	Hydroxy-carboxylic acid receptors
5-HIAA	5-hydroxyindole-acetic acid
3-HK	3-hydroxykynurenine
5-HT	5-hydroxytryptamine, serotonin
HtrA-1	High temperature requiring protein A-1
IDO	Indoleamine-2,3-dioxygenase
IFN-γ	Interferon-γ
IL-	Interleukin-
IL4i1	Interleukin-4-induced protein-1
I3PA	Indole-3-propionic acid
I3PyA	Indole-3-pyruvic acid
JNK	Jun-N-terminal kinases
KAT	Kynurenine aminotransferase
KMO	Kynurenine-3-mono-oxygenase
KP	Kynurenine pathway
K/Q ratio	Kynurenic acid/quinolinic acid ratio
K/T ratio	Kynurenine/tryptophan ratio
LAT-1	Large Neutral Amino Acid Transporter-1
LPS	Bacterial lipopolysaccharides
MDD	Major depressive disorder
MRP4	Multidrug resistance associated protein 4
NAD	Nicotinamide adenine dinucleotide (NAD+)
NMDA	N-methyl-D-aspartate
OAT	Organic Anion Transporter
PAH	*Para*-aminohippuric acid
PBMCs	Peripheral blood mononuclear cells
PFC	Prefrontal cortex
PSA	Prostate Specific Antigen
Ro61-8048	3,4-dimethoxy-N-[4-(3-nitrophenyl)thiazol-2-yl]benzenesulphonamide
TDO	Tryptophan-2,3-dioxygenase
TGF-β1	Transforming Growth Factor-β1
TLR	Toll-Like Receptors
TNF	Tumour Necrosis Factor-α
